# Comparative Genome-Wide Analysis of the Malate Dehydrogenase Gene Families in Cotton

**DOI:** 10.1371/journal.pone.0166341

**Published:** 2016-11-09

**Authors:** Muhammad Imran, Kai Tang, Jin-Yuan Liu

**Affiliations:** Laboratory of Plant Molecular Biology, Center for Plant Biology, School of Life Sciences, Tsinghua University, Beijing 100084, China; New Mexico State University, UNITED STATES

## Abstract

Malate dehydrogenases (MDHs) play crucial roles in the physiological processes of plant growth and development. In this study, 13 and 25 MDH genes were identified from *Gossypium raimondii* and *Gossypium hirsutum*, respectively. Using these and 13 previously reported *Gossypium arboretum* MDH genes, a comparative molecular analysis between identified MDH genes from *G*. *raimondii*, *G*. *hirsutum*, and *G*. *arboretum* was performed. Based on multiple sequence alignments, cotton *MDHs* were divided into five subgroups: mitochondrial MDH, peroxisomal MDH, plastidial MDH, chloroplastic MDH and cytoplasmic MDH. Almost all of the *MDHs* within the same subgroup shared similar gene structure, amino acid sequence, and conserved motifs in their functional domains. An analysis of chromosomal localization suggested that segmental duplication played a major role in the expansion of cotton MDH gene families. Additionally, a selective pressure analysis indicated that purifying selection acted as a vital force in the evolution of MDH gene families in cotton. Meanwhile, an expression analysis showed the distinct expression profiles of *GhMDHs* in different vegetative tissues and at different fiber developmental stages, suggesting the functional diversification of these genes in cotton growth and fiber development. Finally, a promoter analysis indicated redundant but typical *cis*-regulatory elements for the potential functions and stress activity of many MDH genes. This study provides fundamental information for a better understanding of cotton MDH gene families and aids in functional analyses of the MDH genes in cotton fiber development.

## Introduction

Plant malate dehydrogenase (MDH, EC1.1.1.37) possesses multiple isoforms, and catalyzes the interconversion of malate and oxaloacetate (OAA) coupled to the reduction-oxidation of the NAD pool [1]. NAD-dependent MDHs with discrete kinetic properties and physiological functions are located in the cytosol, plastid, mitochondria, peroxisomes and chloroplasts [1, 2]. In general, MDHs are stable as dimers or tetramers, with subunit molecular weights ranging from 30.01 to 35.01 kDa. MDH from *Nitzschia alba* is the only octameric MDH reported so far and is composed of eight identical subunits [3]. Each subunit contains a conserved NAD-binding site (glycine motif) in the dinucleotide NAD-binding domain and a substrate-binding site (H-site/ active site) located in the catalytic C-terminal domain [4]. Based on gene organization and protein sequence similarity, different MDHs have different reaction requirements, malate selectivities and subcellular localizations [4]. To date, abundant MDH genes have been characterized from several plant species, including wheat [5], *Arabidopsis* [6], maize [7], apple [8] and cotton [9]. Among these MDH genes, the mitochondrial and cytosolic MDH genes are the most frequently investigated, likely because of their abundance in the plant kingdom. An increasing number of studies indicate that MDHs and their catalytic product malate are involved in several processes of plant growth and development, e.g., root growth [10], seed development [11], and leaf respiration [6]. MDHs also play crucial roles in various biotic and abiotic stresses, such as pathogen [12], nutrient [9], salt and cold [8] stresses.

A comparison of protein profiles analysis in *Gossypium hirsutum* revealed that MDH is a vital enzyme involved in the regulation of cotton fiber elongation [13]. The accumulation of its product malate in vacuoles was suspected to enhance the turgor pressure, driving fiber elongation [14]. Cell turgor is achieved through the influx of water, which is driven by the osmotically active solute malate [15]. Additionally, the expression and activity of phosphoenolpyruvate carboxylase (PEPC), the enzyme responsible for malate synthesis, correlate with fiber elongation [16]. Thus, MDHs might participate directly in the accumulation of malate at elongation and may play a crucial role in the regulation of fiber elongation.

Cotton is an important economic crop and a major source of natural textile fiber worldwide [17]. The cotton genus (*Gossypium*) comprises approximately 52 species, and *Gossypium hirsutum* (AADD), an allotetraploid species, is the most valuable fiber crop [18, 19], accounting for more than 90% of the world’s cotton production [20]. Allotetraploid cotton is thought to have formed via a natural allotetraploidization event that occurred 1–2 million years ago (MYA) [21] between the *Gossypium arboretum* (AA) as the maternal parent and *Gossypium raimondii* (DD) as the pollen-providing agent [22]. In our previous study of *G*. *arboretum*, the expression profiles of 13 *GaMDH* genes offered us some clues regarding functional diversity [23]. However, little is known about this gene family in allotetraploid cotton, especially the expansion pattern, molecular evolution, subcellular localization and functional diversification of these genes.

The recently published genomic data on *G*. *hirsutum* [24, 25], together with those on *G*. *arboretum* [20] and *G*. *raimondii* [26], provide an opportunity to perform a comparative analysis of the MDH gene family in three cotton species. Here, we initially identified the MDH genes from *G*. *hirsutum* (*GhMDHs*) and *G*. *raimondii* (*GrMDHs*), and then conducted a phylogenetic analysis to classify *GaMDH* genes into subgroups. Furthermore, the resulting classification, orthologous relationships, sequence characteristic, gene structure, conserved motif compositions, expression profiles and *cis*-regulatory element analysis will be useful for future functional studies of the MDH gene family. Our results provide valuable information regarding the MDH genes in cotton, and allow for a better understanding of the evolutionary relationships between these genes among three cotton species.

## Materials and Methods

### Plant materials and growth conditions

*G*. *hirsutum* cultivar ‘CRI35’ was used to examine the expression patterns of MDH genes. The seeds were germinated and maintained in pots under standard conditions for 20 days to collect leaf, stem and root samples. Then, the remaining plantlets were transplanted to an open field at Tsinghua University in China to continue growing. Cotton flowers were tagged on the day of anthesis, and developing ovules were harvested at different developmental stages from 0 to 25 days post anthesis (DPA). The collected samples were immediately frozen in liquid nitrogen and stored at -80°C for nucleic acid extraction.

### Database search for cotton *MDH* genes

Genomic databases of *G*. *raimondii* and *G*. *hirsutum* were obtained from the CottonGen website (http://www.cottongen.org) [24–26]. The identified MDH protein sequences from *G*. *arboretum* and *Arabidopsis* (retrieved from *Arabidopsis* information resource; http://www.arabidopsis.org/) were used as queries in BLASTP searches [27] against the *G*. *raimondii* and *G*. *hirsutum* genome databases. All of the output genes with E-values <1.0 were selected, and redundant sequences were removed manually. Then, all of the identified MDH genes were subjected to the InterProScan (http://www.ebi.ac.uk/interpro/search/sequence-search) to confirm the presence of the MDH domain [28]. The SMART and Pfam databases were used to analyze each member of MDH gene family. Finally, the physicochemical parameters of the full-length cotton MDH proteins were calculated by ExPASy (http://cn.expasy.org/tools). The subcellular localization of each MDH protein was predicted using WoLF PSORT (http://www.genscript.com/wolf-psort.html), TargetP (www.cbs.dtu.dk/services/TargetP) and Predotar (https://urgi.versailles.inra.fr/predotar/test.seq) [29].

### Sequence alignment, phylogenetic tree construction, motif detection and functional divergence analyses

An alignment of all of the full-length MDH proteins was performed using Clustal W with standard settings [30] and confirmed by the MUSCLE [31]. A neighbor-joining phylogenetic tree was constructed by MEGA 6.0 [32], with P-distance and pairwise gap deletion parameters engaged. To assess the statistical reliability of each node, a bootstrap test was conducted with 1000 replicates. To validate the results from the NJ method, a Maximum parsimony tree was constructed using MEGA 6.0 and PHYLIP software [33], and more than 87% of the *GrMDH*, *GaMDH* and *GhMDH* genes were placed in the same position as those in the NJ tree. Meanwhile, the tree topology of the Maximum Likelihood and Minimal Evolution method of MEGA 6.0 was very similar to that of the NJ tree. The protein sequences that were used for phylogenetic tree analysis are presented in S1 Table.

The program DNASTAR Lasergene (http://www.dnastar.com/) was used to calculate the sequence identities of MDHs at both the nucleotide and amino acid levels. The exon-intron structures of the MDH genes were deduced by the alignment of their coding sequences to the representative genomic sequence information obtained from the aforementioned genome databases using the online tool Gene Structure Display Server (http://gsds.cbi.pku.edu.cn) [34].

The deduced MDH protein sequences of three cotton species were submitted to the online Multiple Expectation Maximization for Motif Elicitation (MEME) version 4.11.1 (http://meme-suite.org/tools/meme) for the detailed identification of conserved motifs [35]. The parameters employed in the analysis were as follows: zero or one occurrence per sequence, optimum motif widths ranging from 6 to 60 amino acids and a maximum of 10 motifs.

### Chromosomal localization and duplication of cotton MDH genes

The chromosomal localization of each MDH gene in *G*. *raimondii* and *G*. *hirsutum* was deduced based on the available genomic information from the CottonGen database. The distribution of MDH genes on the chromosomes and their orthologous relationships were visualized using the program Circos [36]. The cotton MDH gene duplication events were investigated using the following criteria: 1) genes with >70% coverage of the alignment length; 2) genes with >70% identity in the aligned region; and 3) a minimum of two duplication events were considered for strongly connected genes [37]. Wei et al. proposed that coparalogous genes located on duplicated chromosomal blocks were considered segmental duplication [38].

Subsequently, all of the protein sequences and the corresponding ORFs of the gene pairs were aligned, and the *Ka* (nonsynonymous substitution rates) and *Ks* (synonymous substitution rates) of the duplicated cotton *MDH* genes were determined by the program *KaKs* Calculator [39]. The average *Ks* values were estimated for each duplicated gene pair, and the conserved flanking protein-coding genes were also used to estimate dates of the segmental duplication events [40]. The time of duplication and deviation of the MDH gene pairs was calculated using the equation T = *Ks*/2λ, assuming clock-like rates of (λ) 1.5 × 10^−8^ substitutions per synonymous site per year for cotton [41]. Eventually, the selection pressure for each gene pair was assessed based on the *Ka/Ks* ratio.

### Promoter sequence analysis

To analyze the promoter, the 1500 bp genomic DNA sequences upstream of the initiation codon (ATG) of each cotton MDH genes was extracted from the genome database. Then, the *cis*-regulatory elements of each promoter sequences were predicted by searching in PlantCARE database (http://bioinformatics.psb.ugent.be/webtools/plantcare/html/) [42].

### Dataset-based gene expression analysis

The RNA-sequencing (RNA-seq) data were downloaded from the National Center for Biotechnology Information Short Read Archive (http://www.ncbi.nlm.nih.gov/sra) with primary accessions code PRJNA248163 and referenced accessions code SRX202873 to generate the expression profiles of *GhMDHs* among different organs and fiber developmental stages [24]. The fragments per kilobase of exon model per million fragments mapped (FPKM) was used to normalize these data. Finally, log2-transformed FPKM values from 11 tissues were used to construct a heatmap.

### RNA isolation and quantitative RT-PCR analysis

The total RNAs of all the collected samples were extracted using the RNAprep Pure Plant kit (TIANGEN, Beijing, China). A total of 2 μg of RNA was used as the template, and the first-strand cDNAs were synthesized using the Takara Reverse Transcription System (TaKaRa, Shuzo, Otsu, Japan). The resulting cDNA products were diluted 1/5 and stored at -20°C for quantitative real-time PCR (qRT-PCR) analysis. Using the specific primers for each *GhMDH* gene (S2 Table), qRT-PCR reactions were performed in a Mini Opticon Real-Time PCR System (Bio-Rad, CA, USA) using the SYBR Green Master Mix Reagent (TaKaRa, Shuzo, Otsu, Japan) 20 μL according to the manufacturer’s protocol. Cotton *UBQ7* was used as an internal reference gene, and three biological replicates were performed for each sample. The thermal cycling conditions were as follows: pre-denaturation at 95°C for 5 min and 40 cycles of amplification at 95°C for 5 s, 58°C for 30 s and 70°C for 30 s. The comparative 2^-ΔΔCT^ method was used to calculate the relative expression levels [43]. The heatmap for the gene expression profiles was generated with Mev 4.0 (http://www.tm4.org/) [44].

## Results

### Identification and classification of cotton MDH genes

Through a systematic BLAST search against the *G*. *raimondii* and *G*. *hirsutum* genome databases with query sequences of *G*. *arboretum* and *Arabidopsis* MDH proteins, the candidate MDH genes were identified. Then, all of these retrieved sequences were verified by Pfam and InterProScan analyses. A total of 13 and 25 non-redundant genes containing both a typical MDH dinucleotide NAD-binding domain and a catalytic C-terminal domain were confirmed in the *G*. *raimondii* and *G*. *hirsutum* genomes, respectively (Table 1). The properties of newly identified *Gossypium* MDHs were analyzed by ExPASy. More than 89% of the 38 MDHs encoded proteins ranging from 300 to 440 amino acids, whereas four (one from *G*. *raimondii* and three from *G*. *hirsutum*) had clearly different lengths compared to the MDH homologs reported in the genomic analysis of *G*. *arboretum* [23], *i*.*e*., they were less than 300 or more than 500 amino acids. The nucleotide lengths of these MDHs ranged from 771 to 1692 bp, and their molecular weights and theoretical p*I* values ranged from 27.26 kDa to 59.78 kDa and 5.27 to 6.37, respectively. Subcellular localization is crucial for understanding the functional involvement of genes [45]. In this study, WoLF PSORT, TargetP and Predotar were used for primary structural analyses of cotton malate dehydrogenases. The results showed that most of the 25 *G*. *hirsutum MDHs (GhMDHs)* and 13 *G*. *raimondii MDHs (GrMDHs)* genes possessed signal sequences and were predicted to be located in the plastid, mitochondria and cytoplasm; only some genes were predicted to be located in the peroxisomes and chloroplast (Table 1). Moreover, the predicted subcellular localization results showed more than 90% homology predictions with each method.

**Table 1:** The MDH genes in *G*. *raimondii* and *G*. *hirsutum* and their sequence characteristics.

Gene name	Gene Identifier	Genome position	CDS	Proteins			Subcellular location
				Size(aa)	MW(kDa)	*p*I	
*GrmMDH1*	Gorai.009G372600.1	Chr09 (+): 50437387–50440554	1017	338	35.34	8.62	Mitochondria
*GrmMDH2*	Gorai.002G063300.1	Chr02(-): 7415313–7418604	1020	339	35.46	8.63	Mitochondria
*GrmMDH3*	Gorai.004G030200.1	Chr04(-): 2392452–2395553	606	201	21.14	8.83	Mitochondria
*GrpxMDH1*	Gorai.002G211700.1	Chr02(-): 55893406–55895928	1062	353	37.18	7.22	Peroxisomes
*GrpxMDH2*	Gorai.003G034300.1	Chr03(+): 3412885–3416117	1080	359	37.92	7.61	Peroxisomes
*GrpdMDH1*	Gorai.003G097400.1	Chr03(-): 30183409–30186613	1239	412	43.22	8.19	Plastid
*GrpdMDH2*	Gorai.001G074600.1	Chr01(+): 7631996–7634982	1239	412	43.48	8.44	Plastid
*GrpdMDH3*	Gorai.007G037800.1	Chr07(-): 2612485–2614471	1299	432	46.06	8.29	Plastid
*GrchMDH*	Gorai.012G007300.1	Chr12(-): 837686–842873	1317	438	47.95	6.75	Chloroplast
*GrcMDH1*	Gorai.005G050200.1	Chr05(-): 4944349–4947387	999	332	35.51	6.35	Cytoplasm
*GrcMDH2*	Gorai.011G174200.1	Chr11(-): 39503476–39507273	1002	333	35.72	6.6	Cytoplasm
*GrcMDH3*	Gorai.008G107500.1	Chr08(-): 33700522–33702402	1001	333	36.04	6.91	Cytoplasm
*GrcMDH4*	Gorai.006G221400.1	Chr06(+): 47414606–47416610	1106	368	40.67	5.66	Cytoplasm
*GhmMDH1A*	Gh_A04G0320	ChrA04(-): 7659308–7661994	1017	338	35.44	8.78	Mitochondria
*GhmMDH1D*	Gh_D05G3328	ChrD05(+): 53461998–53464635	1017	338	35.37	8.63	Mitochondria
*GhmMDH2A*	Gh_A01G0404	ChrA01(-): 6392538–6395256	1020	339	35.49	8.78	Mitochondria
*GhmMDH2D*	Gh_D01G0410	ChrD01(-): 4837470–4840091	1053	339	35.46	8.63	Mitochondria
*GhmMDH3A*	Gh_A08G0191	ChrA08(-): 1924683–1927435	849	282	29.63	6.04	Mitochondria
*GhmMDH3D*	Gh_D08G0268	ChrD08(-): 2556634–2561081	771	256	27.26	5.27	Mitochondria
*GhpxMDH1A*	Gh_A01G1505	ChrA01(-): 90792502–90794567	1062	353	37.25	6.75	Peroxisomes
*GhpxMDH1D*	Gh_D01G1750	ChrD01(-): 54261419–54263478	1062	353	37.23	6.76	Peroxisomes
*GhpxMDH2A*	Gh_A02G1418	ChrA02(-): 79906521–79909050	1080	359	38.07	7.61	Peroxisomes
*GhpxMDH2D*	Gh_D03G0301	ChrD03(+): 3394327–3398640	1692	563	59.78	6.37	Peroxisomes
*GhpdMDH1A*	Gh_A03G0590	ChrA03(-): 15570877–15572115	1239	412	43.17	7.65	Plastid
*GhpdMDH1D*	Gh_D03G0874	ChrD03: 30208751–30209989	1239	412	43.27	8.19	Plastid
*GhpdMDH2A*	Gh_A07G0596	ChrA07(+): 8259423–8260661	1239	412	43.37	8.25	Plastid
*GhpdMDH2D*	Gh_D07G0663	ChrD07(+): 7736819–7738057	1239	412	43.48	8.44	Plastid
*GhpdMDH3A*	Gh_A11G0288	ChrA11(-): 2671112–2672335	1224	407	43.09	8.01	Plastid
*GhpdMDH3D*	Gh_D11G0345	ChrD11(-): 2932410–2933633	1224	407	43.01	8.79	Plastid
*GhchMDHA*	Gh_A05G3552	ChrA05(+): 91232469–91237197	1320	439	48.00	6.75	Chloroplast
*GhchMDHD*	Gh_D04G0053	ChrD04(-): 806012–810692	1320	439	48.03	6.54	Chloroplast
*GhcMDH1A*	Gh_A02G0386	ChrA02(-): 4867855–4870199	999	332	35.55	6.35	Cytoplasm
*GhcMDH1D*	Gh_D02G0438	ChrD02(-): 5773634–5776021	999	332	35.51	6.35	Cytoplasm
*GhcMDH2A*	Gh_A10G2294	Scaf.2503_A10(-): 57412–60654	1002	333	35.66	6.6	Cytoplasm
*GhcMDH3A*	Gh_A12G0868	ChrA12(-): 57778473–57780379	1002	333	35.96	6.91	Cytoplasm
*GhcMDH3D*	Gh_D12G0949	ChrD12(-): 34802283–34803999	1002	333	36.04	6.3	Cytoplasm
*GhcMDH4A*	Gh_A09G1819	ChrA09(+): 71548871–71551715	1029	342	37.38	6.04	Cytoplasm
*GhcMDH4D*	Gh_D09G1940	ChrD09(+): 46873204–46874532	936	311	33.81	8.07	Cytoplasm

**Fig 1:**
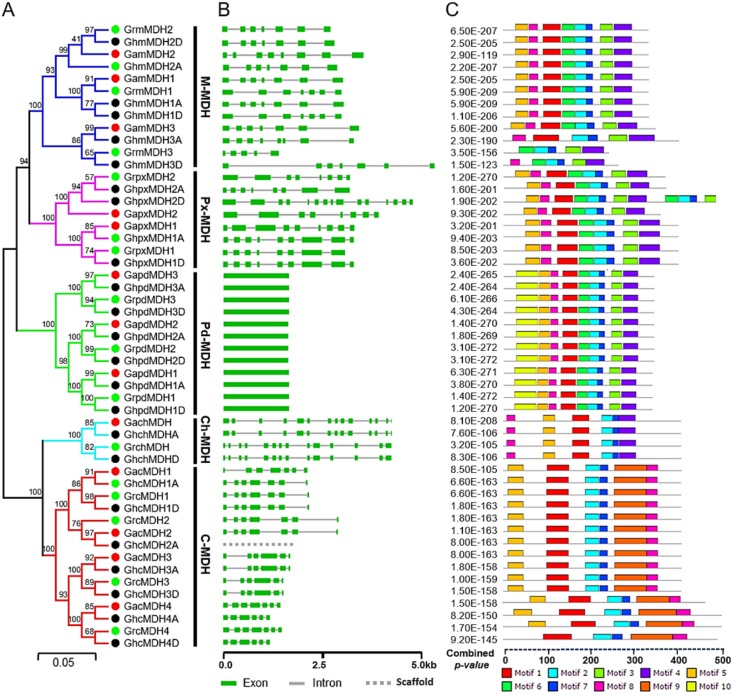
Phylogenetic relationships, gene structure and conserved motif analysis of MDH genes in *G*. *arboretum*, *G*. *raimondii*, and *G*. *hirsutum*. A. The unrooted phylogenetic tree was constructed using MEGA 6.0 with the Neighbor-Joining method, and a bootstrap analysis was performed with 1000 replicates. The MDH genes from *G*. *arboretum*, *G*. *raimondii*, and *G*. *hirsutum* were highlighted in red, green and black dots, respectively. The branches of each subgroup are presented in a specific color. M-MDH, Px-MDH, Pd-MDH, Ch-MDH, and C-MDH indicate mitochondrial, peroxisomal, plastidial, chloroplastic and cytoplasmic MDH subgroup, respectively. B. The exon-intron structures of MDH genes from *G*. *arboretum*, *G*. *raimondii*, and *G*. *hirsutum*. The green boxes, and the gray line indicates exons and introns. C. Different motifs are indicated in different colors. The regular expression and sequences of motifs 1–10 are listed in S3 Table.

To determine the evolutionary relationship between and confirm the classification of MDH genes, all the putative *MDHs* from three cotton species were aligned to generate an unrooted phylogenetic tree using the Neighbor-joining method (Fig 1A). The phylogenetic tree divided the MDH gene family of *G*. *raimondii*, *G arboretum* and *G*. *hirsutum* into five subgroups based on sequence similarities and sequences conserved for subcellular localization, *i*.*e*., mitochondrial *MDHs* (*M-MDHs*), peroxisomal *MDHs* (*Px-MDHs*), plastidial *MDHs* (*Pd-MDHs*), chloroplastic *MDHs* (*Ch-MDHs*) and cytoplasmic *MDHs* (*C-MDHs*). Comparative analyses of the phylogenetic tree suggested that the classification of the *G*. *raimondii* and *G*. *hirsutum* MDH gene family was almost similar and applicable to the *G*. *arboretum* MDH gene family. According to the phylogenetic relationships with orthologs in *G*. *arboretum* and based on subcellular localization, 13 *GrMDHs* were named as *GrmMDH1* to *GrmMDH3*, *GrpxMDH1* to *GrpxMDH2*, *GrpdMDH1* to *GrpdMDH3*, *GrchMDH*, and *GrcMDH1* to *GrcMDH4* (Fig 1A and Table 1). Using the gene location and orthologous relationships, we designated 25 *GhMDHs* as *GhmMDH1A/1D* to *GhmMDH3A/3D*, *GhpxMDH1A/1D* to *GhpxMDH2A/2D*, *GhpdMDH1A/1D* to *GhpdMDH3A/3D*, *GhchMDHA/D* and *GhcMDH1A/1D* to *GhcMDH4A/4D* (Fig 1A and Table 1). Altogether, each diploid progenitor had its own ortholog in allotetraploid, with the exception of *GrcMDH2*. Subsequently, after repeated genome scanning and amplification using specific primers, we still did not find a putative *GhcMDH2D*. Thus, we concluded that it might have been lost during the evolution of *G*. *hirsutum*. Among these cotton *MDH* subgroups, *C-MDHs* constituted the largest subgroup, with 15 members. The second and third largest subgroups (*M-MDHs* and *Pd-MDHs*) each consisted of 12 members. In subgroup *Px-MDHs* and *Ch-MDHs*, eight and four members were identified, respectively (Fig 1A). Overall, the phylogenetic tree analysis revealed a highly conserved amino acid sequence, suggesting a strong evolutionary relationship within members of each subgroup of cotton *MDHs*.

### Sequence features of cotton MDH genes

To examine the sequence characteristics of the MDH genes in three cotton species, we calculated the sequence identities at the nucleotide and amino acid level of individual *MDH* in terms of their phylogenetic relationship to provide valuable clues concerning the evolution of specific gene families (S1A Fig). The results showed that the percentage of sequence identities of all the genes was higher within the groups, particularly for those with an orthologous relationship. Similarly, the *M-MDH*, *Pd-MDH* and *C-MDH* subgroups had a higher sequence identity across the group due to a close evolutionary distance (S1A Fig). Importantly, three gene pairs (*mMDHs1/2* (*GamMDH1/2*, *GrmMDH1/2*, *GhmMDH1A/2A* and *GhmMDH1D/2D*), *pdMDHs 1/2*, and *cMDHs 1/2*) with more than 90% identity existed in three subgroups, indicating that they might have originated from gene duplication events (S1 Fig).

To further explore the structural diversity of MDH genes, we compared 51 *MDH* cDNAs to their genomic sequences and revealed that the number of exons varied from 1 (member of subgroup *Pd-MDH*) to 14 (member of subgroup *Ch-MDH*). A detailed illustration of the exon-intron structure is shown in Fig 1B. More than 65% of the MDH genes (35 out of 51) contained approximately 8 exons, which seems to be a common feature of the MDH gene family. Similarly, a total of 297 introns were found in the cotton MDH genes, with an average intron number of 7.2 per gene and an average intron length of 253.4 bp. More concisely, the high similarity levels of the exon-intron organization and the phylogenetic relationships between the MDH genes within each subgroup suggested gene structure conservation, thus supporting the close evolutionary relationships of cotton *MDHs* and the classification of five groups (*M-MDHs* to *C-MDHs*).

**Fig 2:**
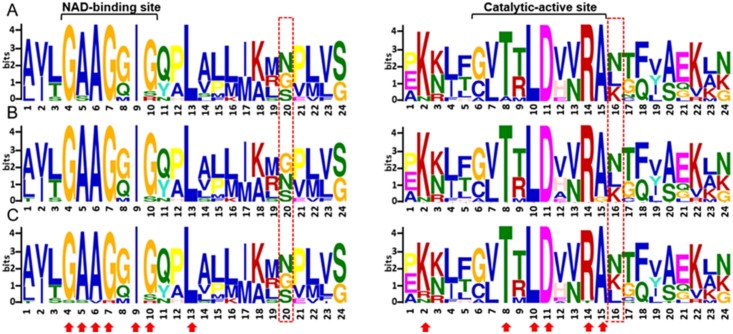
A conserved glycine motif and a catalytic active site were present in *G*. *arboretum* (A) *G*. *raimondii* (B) and *G*. *hirsutum* (C). The highly conserved amino acids are indicated by *arrows*. Two inversions of the second and third conserved residue are marked a *rectangle*. The sequence position of each domain and the information content measured in bits are shown on the x and y axes, respectively.

To expose the diversification of MDH proteins, a MEME analysis was also performed. As shown in Fig 1C, a total of 10 conserved motifs designated as motif 1 to motif 10 were identified (S3 Table and S2 Fig). The cotton MDH genes within the same groups contained similar motifs, of which more than 45% were conserved among all the genes. All of the members of the M-MDH group contained the most-conserved motifs, with the largest numbers 1~8 and 10, while most of the C-MDH and Ch-MDH group members possessed the lowest motifs, 1, 2, 5 and 7~9 (Fig 1C). By comparison, the members of the Pd-MDH and Px-MDH subgroups contained an intermediate number, 1~8. Moreover, the predicted motifs were further annotated by ScanProsite [46], and only two motifs (motif 2 and motif 5) could be matched to the annotated motifs in the database. Motif 1 and motif 5 were shared among all of the cotton MDHs except for GrmMDH1, GhmMDH3D, GhmMDH3A and GhcMH4D. These motifs are represented within the dinucleotide NAD-binding domain, which had important conserved arginine residues (Arg^106^, Arg^112^, and Arg^178^) and a glycine motif (GXXGXXG) specific to substrate binding and was significant in structure stabilization [47]. The variations in the sequences of these motifs exhibited different binding affinities [48]. The conserved motif 2, motif 7 and motif 8 contained the catalytic pair (Asp^175^ and His^202^) within the major catalytic C-terminal domain, which is required to enhance the rate of catalysis [49]. More interestingly, we also found that GhpxMDH2D from subgroup Px-MDH possessed domain repeats (S1B Fig). In general, domain repeats are thought to have evolved through intragenic duplication and recombination events [50], and the formation of a new domain is an essential mechanism that allows an organism to enhance its cellular functions and activity [51, 52]. The presence of MDH catalytic domain repeats contributes to the complexity of this gene family and may play a crucial role in MDH gene evolution. The comparative analysis of conserved motif suggested that protein functions have been both conserved and diverged during the evolution of the MDH gene family. To further reveal the distinctive domain features of three cotton species, comparative analyses for the conservation of amino acid residue in functional domains were performed and presented by MEME (Fig 2 and S1B Fig). The results showed that some amino acids were extremely conserved (indicated by arrow), while, for others, there was a certain degree of variation. Comparing the glycine motif and catalytic domain in three cotton species, most of the vital residues were highly conserved, except for two inversions of the second and third conserved residues at positions 20 and 16 (indicated by a rectangle), respectively (Fig 2).

### Chromosomal distribution and duplication analysis of cotton MDH genes

**Fig 3:**
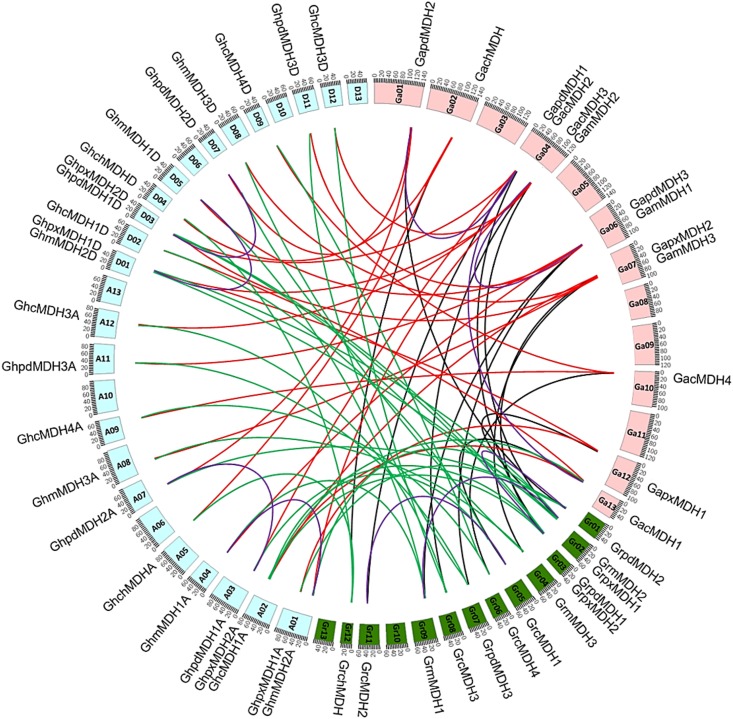
Orthologous MDH genes pairs identified between *G*. *arboretum*, *G*. *raimondii* and *G*. *hirsutum*. The chromosomes of *G*. *arboretum* (Ga1-Ga13), *G*. *raimondii* (Gr1-Gr13), and *G*. *hirsutum* (A1-A13 and D1-D13) are filled with light peach, green and ice blue color, respectively. The orthologous MDH genes are connected by black, red and green lines, while segmental duplicated MDH genes are connected by purple line. The duplicated gene pairs and orthologous relationships between the genomes are represented by Circos figure.

To further investigate the relationship between the genetic divergence of the MDH gene family and gene duplication, all of the cotton MDH genes were mapped to their corresponding chromosomes. Of 51 MDH genes, 50 were present on the chromosomes of *G*. *raimondii*, *G*. *arboretum* and *G*. *hirsutum* (Fig 3). Normally, one or two members were unevenly distributed on most chromosomes, with the exception of *G*. *arboretum*, in which chromosome 4 contained four genes. For the *GhMDH* gene family, 24 out of 25 *MDHs* were allotted up to 20 of the 26 *G*. *hirsutum* chromosomes, and the remaining gene, *GhcMDH2A* showed affinity with yet unmapped scaffolds (Fig 3). For the *GrMDH* gene family, all 13 *MDHs* were allotted to 11 of the 13 *G*. *raimondii* chromosomes (Fig 3). Genomic changes, including chromosomal rearrangements and gene duplication, play a significant role in the generation of gene families [53]. To elucidate the expanded mechanism of the MDH gene family in *G*. *raimondii*, the gene duplication events were investigated, and 3 duplication events, *GrcMDH1/GrcMDH2*, *GrpdMDH1/GrpdMDH2* and *GrmMDH1/GrmMDH2*, occurred from 18.59 to 19.53 MYA, which is consistent with the timing of a large-scale genome duplication in cotton [25]. Similarly, in *G*. *hirsutum*, four segmental duplication events, *GhpdMDH1A/GhpdMDH2A*, *GhmMDH1A/GhmMDH2A*, *GhpdMDH1D/GhpdMDH2D* and *GhmMDH1D/GhmMDH2D*, jointly took place during from 19.069~20.668 MYA (Fig 3 and S4 Table). Interestingly, all of the segmentally duplicated gene pairs were separated into different gene clusters, which suggest that the expansion of the MDH gene family in cotton was mainly attributed to segmental duplication events [19].

To explore the selective constraints on duplicated MDH genes, the *Ka/Ks* ratio was estimated for all 10 duplicated MDH gene pairs. Usually, *Ka/Ks* > 1 indicates positive selection; *Ka/Ks* = 1 indicates neutral evolution; and *Ka/Ks* < 1 indicates purifying selection [54]. In this study, 3 duplicates pairs in the MDH gene family of *G*. *raimondii*, 3 duplicate pairs in *G*. *arboretum* and 4 duplicated pairs in *G*. *hirsutum* were investigated. The results demonstrated that all of the duplicated gene pairs had a *Ka/Ks* ratio of less than 0.3, and no significant differences were found among different cotton species, suggesting that these species experienced strong purifying selective pressure (S5 Table). These observations indicate that the functions of the duplicated genes in three cotton species did not diverge much and that purifying selection might significantly contribute to the maintenance of function in the cotton MDH gene family. Therefore, from our results, it also could be speculated that the amplification of the MDH gene family was mainly caused by segmental duplication events and that purifying selection has dominated across duplicated genes in *G*. *hirsutum* (S5 Table).

### Expression profiles of *GhMDH* genes

Expression profiling is a feasible tool for understanding gene function. To survey the expression patterns of upland cotton MDH genes in different tissues/organs, we tested the spatial-specific expression patterns of *GhMDHs* by performing quantitative RT-PCR and RNA-sequencing data analysis. Due to the greatest resemblance between the mRNAs of the *GhMDHxA*-*GhMDHxD* gene pairs, we regarded them as one combination named *GhMDHx* and checked the expression level by quantitative RT-PCR. Then, we distinguished the portion of *GhMDH1A* from that of *GhMDH1D* by analyzing RNA-seq data. Furthermore, using RNA-seq data analysis, the expression patterns of *GhMDHs* obtained from qRT-PCR and semi-quantitative PCR were confirmed.

**Fig 4:**
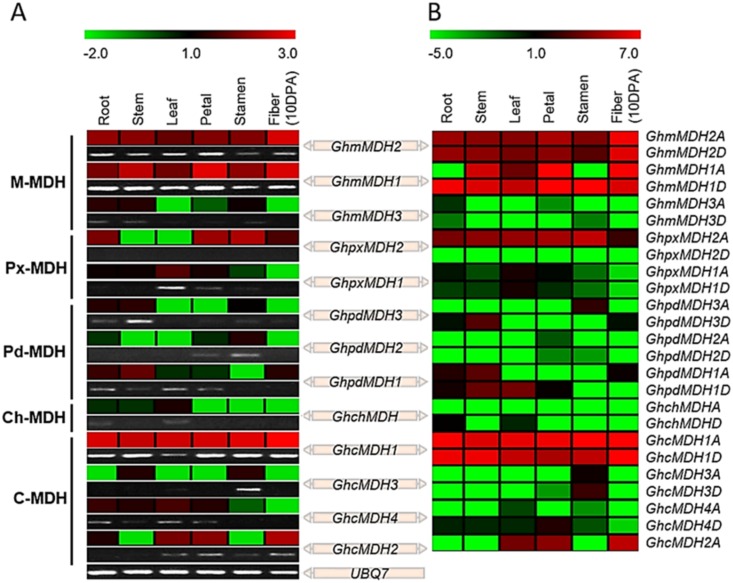
Expression patterns of *GhMDHs* in vegetative tissues of *G*. *hirsutum*. A. Quantitative RT-PCR and semi-quantitative PCR results. B. RNA sequence data analysis results. Sequence Read Archive available under accession numbers SRX797899, SRX797900, SRX797901, SRX797903, SRX797904 and SRX797918 were retrieved from the public repository database for expression analysis in cotton roots, stems, leaves, petals, stamens and 10 DPA fibers, respectively. The color bar in the top of the left column of the heat map represents the relative signal intensity, while that in the top of right column indicates the FPKM-normalized log^2^ transformed counts.

The results showed that all of the *GhMDHs* were differentially expressed in different cotton tissues under normal growth conditions. Among all of the 13 analyzed genes, *GhcMDH1* had the most prominent expression levels in all tissues, followed by *GhmMDH1* and *GhmMDH2* (Fig 4). These high expression levels indicate the important role of these genes in all of these plant tissues. The expression of *GhcMDH3* was detected only in the stamen, and *GhcMDH2* showed the second highest expression level in almost all of the cotton tissues, except for the stem and stamen, and belongs to the largest subgroup, *C-MDH*. *GhpxMDH2* from the *Px-MDH* subgroup expressed the third highest levels. Similarly, *GhpxMDH1*, *GhpdMDH1*, *GhpdMDH3* and *GhcMDH4*, from the *Px-MDH*, *Pd-MDH*, and *C-MDH* subgroups, respectively, were expressed at moderate levels. As shown in the Fig 4A, expression analyses of *GhpdMDH2*, *GhchMDH*, and *GhmMDH3*, showed an almost undetectable expression in all of the tissues with few exceptions. Furthermore, the expression data analysis of the identified paralogous pairs of *GhMDH* genes in 11 cotton tissues revealed a high level of expression conservation. For example, *GhmMDH1/GhmMDH2* and *GhcMDH1/GhcMDH2* showed similar patterns of expression. These diverse expression patterns of *GhMDH* genes indicate the functional diversification of this gene family in *G*. *hirsutum*. To gain more insights into the expression pattern of *G*. *hirsutum* MDH genes, we first analyzed an RNA-seq dataset that encompassed results from six studied cotton tissues. In this case, we productively separated the contribution of *GhMDHxAs* from that of *GhMDHxDs*. Overall, the results of the RNA-seq expression data closely agreed with those of quantitative RT-PCR (Fig 4B).

**Fig 5:**
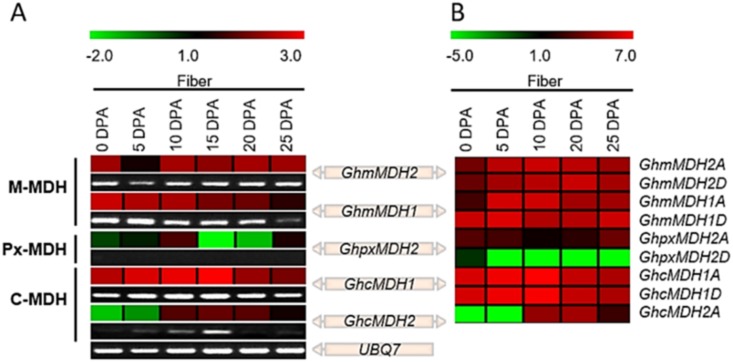
Expression patterns of *GhMDHs* in developing fibers of *G*. *hirsutum*. A. Quantitative RT-PCR and semi-quantitative PCR results. B. RNA sequence data analysis results. Sequence Read Archive available under accession numbers SRX797909, SRX797917, SRX797918, SRX797919 and SRX797920; this information was retrieved from a public repository database for expression analysis in developing fibers (0, 5, 10, 20 and 25 DPA, respectively). The color bar at the top left column of the heat map represents the relative signal intensity, while that at the top right column heat map indicates the FPKM-normalized log^2^ transformed counts.

Next, we also investigated the expression patterns of five *GhMDHs* (*GhmMDH1*, *GhmMDH2*, *GhpxMDH2*, *GhcMDH1* and *GhcMDH2*) at the representative stages of fiber development (0~25 DPA) by quantitative RT-PCR and RNA-seq data analysis (Fig 5). Among the five detected *GhMDHs*, *GhcMDH1* was significantly different from other members; this began to increase in fibers from 5 to 15 DPA and then started to decline, implying that this gene plays a crucial role in fast fiber elongation (Fig 5A and 5B). These results indicate that *GhcMDH1A* and *GhcMDH1D* might jointly play a major role in fiber development, specifically in the elongation stage of fiber. In addition, *GhmMDH1* and *GhmMDH2* may also play an important role in fiber development near the time of fiber initiation (0 to 5 DPA) and secondary cell wall synthesis (20 to 25 DPA; Fig 5A and 5B).

### Putative *cis*-elements in the promoter regions of cotton MDH genes

**Fig 6:**
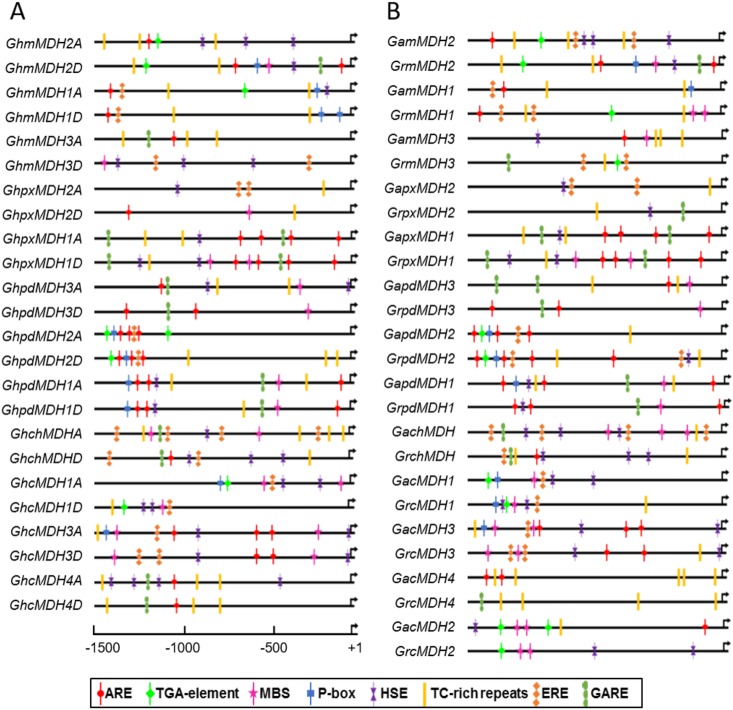
Putative regulatory *cis*-elements in MDH genes promoters from *G*. *hirsutum* (A), *G*. *arboretum* and *G*. *raimondii* (B). The relative positions of *cis*-regulatory elements and transcriptional start sites are shown on the line representing the 1500 bp upstream region of each MDH gene promoters. Only *cis*-elements required for auxin, gibberellic acid, ethylene responses, anaerobic induction, MYB-binding site and defense and heat stress are shown.

The discovery of *cis*-regulatory elements in the promoter region is essential for the elucidation of gene expression pattern [55]. Martin demonstrated that the regulatory sequences of most plant genes resided within a region 500 bp upstream of the start codon [56]. Accordingly, a promoter analysis of the 50 cotton MDH genes using the PlantCARE database revealed that most of the *cis*-regulatory elements were conserved in the 1500 bp upstream of the translational start site (S6 Table). The conservation of *cis*-regulatory elements reflected the importance of the meaningful transcriptional regulation of cotton MDHs. Furthermore, among these highly conserved elements, mostly *cis*-elements were associated with important physiological processes, such as hormone and stress responsiveness (Fig 6 and S6 Table). In previous studies, the activities of many elements have been already confirmed in model Arabidopsis as well as cotton fibers. For instance, ARE, a *cis*-acting regulatory element essential for anaerobic induction, could be identified in 40 MDH gene promoters (S6 Table), suggesting that these genes might be induced by a low oxygen level [57]. MYB transcription factors are involved in the regulation of cotton fiber initiation and elongation [58]. Remarkably, the MBS element (MYB binding site) existed in the promoter regions of 31 cotton MDH genes, and the most preferentially expressed *GhcMDH1A* had two copies (Fig 6), suggesting that this MYB-binding site might direct cotton fiber elongation [58].

Multiple hormones regulate numerous plant growth, developmental processes and response to various stresses [59, 60]. Some previous reports revealed that gibberellic acid (GA) [61], ethylene [62] and auxin [63] may regulate cotton fiber elongation. Interestingly, some cis-elements *i*.*e*., P-box, GARE, ERE and TGA-element (specific for the gibberellic acid, ethylene, and auxin responses) were found in the promoter regions of five different *GhMDH* genes expressed in elongating allotetraploid cotton fibers (Figs 4 and 5). Among the nine *GhMDH* genes expressed in cotton fiber, two highly expressed genes, *GhmMDH1A* and *GhcMDH1A*, had more than three types of phytohormone response elements, whereas *GhpxMDH2A* had less than two type and was the lowest expressed member (Fig 4), suggesting that the regulation of gene expression by these *cis*-regulatory elements could be either positive or negative (Fig 6 and S6 Table). Furthermore, meristem-specific regulation, *i*.*e*., CAT-box, was present only in some *MDHs*, while the presence of *cis*-regulatory elements, such as Skin-1 motif, GCN4 and O2 sites, conferred endosperm-specific gene expression. Finally, stress-responsive elements related to adverse environmental stimuli, such as defense (TC-rich), and heat stress responsiveness (HSE), has also been identified in the promoter regions of mostly cotton MDH genes (Fig 6 and S6 Table).

## Discussion

Previous studies reported that malate is dynamic and correlates with fiber developmental stages, indicating that the regulation of malate dehydrogenase is critical for fiber development [14]. Thus, it is very important to perform a comprehensive analysis of MDH gene families among allotetraploid and diploid cotton species by using available *G*. *raimondii*, *G*. *arboretum* and *G*. *hirsutum* genome sequences.

In this study, a sequence alignment analysis showed that 51 cotton MDH genes were grouped into 2 main phylogenetic group and 5 clear subgroups with distinct subcellular localization. Among these subgroups, mitochondrial MDHs and cytosolic MDHs are two major groups. Recently, the subcellular location of mitochondrial MDH (GhmMDH1) has been determined by Wang et al. [9], meanwhile we have confirmed the subcellular location of cytosolic MDH in the cytosol during the functional characterization of a cotton cytosolic malate dehydrogenase (GhcMDH1) gene (unpublished data). Whereas, the subcellular locations of other orthologues from peroxisomal MDH (AtpMDH1), plastidal MDH (AtpdMDH) and chloroplastic MDH (BrachMDH) have also been confirmed in several functional studies reported by Pracharoenwattana et al. [64], Selinski et al. [65] and Li et al. [66], respectively. These results obviously support our predicted MDHs subcellular locations. More interestingly, the exon-intron structures and motif compositions are well conserved in each subgroup, indicating that the members of the same subgroup might have a common ancestor (Fig 1). Despite specific features of the functional domains near the N-terminus, the sequence identities of the C-terminus were relatively conserved, and more than 89% of all cotton MDHs have exhibited the expected catalytic activity (Fig 2 and S7 Table) [4]. Thus, we hypothesized that all cotton MDHs might have evolved from one original ancestor followed by some unknown changes that took place near the N-terminus, dividing the cotton MDH gene family into five distinct MDH subgroups. Furthermore, by combining the phylogenetic tree and subcellular localization of cotton MDH genes, it is obvious that subgroup C-MDH comprised 7 members and was specific to the cytoplasm, while M-MDH and Pd-MDH subgroups consisted of 6 members each, both were likely directed to the mitochondria and plastid based on localization predictions (Fig 1 and Table 1). In contrast, the remaining members were located in peroxisomes and chloroplasts. Similarly, in *Arabidopsis*, the cMDH1-3, mMDH1-2, pxMDH1-2, and pdMDH1-2 were predicted to be localized to the cytoplasm, mitochondria, peroxisomes, and plastid, respectively, and established an interesting link between the phylogenetic subgroups of MDH proteins and their subcellular localization [64, 65, 67]. Moreover, the sequence identities between cotton MDHs and *Arabidopsis* homologs were greater than 80% with members of each subgroup (data not shown). These results suggest that the localization of MDHs in the same group is relatively conserved among different species and might provide clues as to their specific cellular functions. For example, in 2014, Selinski reported that the plastidial MDH gene is crucial for energy homeostasis in developing *Arabidopsis thaliana* seeds [65]. While, the chloroplastic MDH gene plays an important role in maintaining oxidative stress in *Arabidopsis* plants lacking the malate valve enzyme [68], suggesting that plant malate dehydrogenases evolved various strategies to control their functional activities.

In addition, different physiological functions of plant malate dehydrogenases correlate with their different subcellular localization. For example, *GhmMDH1* and *GhcMDH1* from the mitochondrial MDH and cytoplasmic MDH subgroups, respectively, were highly expressed in vegetative tissues and at different developmental stages of fiber, suggesting that they might play an important function in cotton growth and fiber development. Similarly, Wang et al. (2015) reported that *GhmMDH1*-overexpressing plants had a significantly elevated MDH activity and malate content, whereas RNAi plants showed a lower activity and malate level compared to those of with wild-type plants [9]. In *Arabidopsis*, the MDH genes with decrease leaf respiration and altered photorespiration are located in the mitochondria [6]. Reducing mMDH via antisense inhibition in transgenic tomato plants changes the root architecture and photosynthetic metabolism [69] because mitochondrial MDH oxidizes malate from fumarase reaction to form citrate in the tricarboxylic acid cycle (TCA) and provides reducing equivalents of NAD^+^ for Gly decarboxylases [70]. In contrast, based on kinetic parameters, cytoplasmic MDH is a key enzyme involved in malic acid synthesis [71]. Previous cotton fiber-related malate studies also suggested that malic acid accumulation in the vacuoles may be a major reason for the rapid elongation of the fibers, especially at 15 DPA, and may play an important regulatory role during fast-fiber elongation stages ranged from 5 to 15 DPA [14]. Similarly, the overexpression of apple cytoplasmic MDH increases the expression of proton pump genes, which aids in the transportation of solute and ions into vacuoles by generating an electrochemical gradient and further contributes to cell growth and expansion [71]. Amemiya et al. (2006) reported a similar result in which the knockdown of the vacuolar proton pump vATPase resulted in small tomato fruits [72]. However, in this study, all of the cotton MDH genes were located in their specific subcellular locations (for example GhmMDH1 was located in the mitochondria and *GhcMDH1* in the cytoplasm); thus, the results from Gietl further support our results that *MDH* gene families contain the conserved MDH domain but have an organelle-specific N-terminal transit peptide and specific operational activity that leads to their different localizations and presumably diverse functions [73].

Furthermore, in various genome-wide analyses, the mRNA expression patterns are similar for groups of functionally related genes, even similar within cellular compartments [74, 75]. To assess the extent of this relationship, we compared the expression profiles and the predicted subcellular location of MDH genes in allotetraploid cotton. For example, *GhmMDH1*/*2* and *GhcMDH1*/*2* showed similar expression patterns, but *GhmMDH1* and *GhcMDH1* were highly expressed especially in cotton fiber developmental stages and localized to the mitochondria and cytoplasm (Fig 5 and Table 1). In contrast, the expression profiles of peroxisomal, plastidial and chloroplastic MDHs were lower in all cotton tissues. Thus, Drawid’s study further strengthens our results that the genes/proteins showed the highest level of expression in the cytoplasm and the lowest level of expression in other compartments. More precisely, genes associated with cytoplasmic proteins may be differentially regulated in different dynamic ranges than those associated with mitochondria and other membrane proteins [76]. However, until now, the functional characterization of *GhMDH* genes has remained mostly unknown. Therefore, determining the functional characterization of the *GhcMDH1* gene from the cytoplasmic subgroup is a crucial step in elucidating the function of this gene family in fiber elongation.

Finally, the involvement of *MDHs* in the regulation of abiotic stress has been reported by various studies, for example, the overexpression of cytosolic MDH in *Medicago sativa* led to increased aluminum (Al) tolerance via metal chelation in the soil [77]. Whereas, when cotton is cultured in medium containing only insoluble phosphorus, Ca-phosphorus, or Al-phosphorus, *GhmMDH1* overexpressing plant produced significantly higher biomass and had a longer root and Phosphorus content than wild type plants, however, knockdown plant showed the opposite results [9]. Interestingly, in the promoter region of allotetraploid cotton MDH genes, we found that 38 and 33 genes possessed an average of 1.60 and 1.40 copies per gene for adverse environmental stimulus, such as defense and heat stress responsive elements, respectively (S6 Table and Fig 6). The distribution of these elements was more extensive compared to that of the preferentially expressed genes that contained 1.50 and 1.37 copies per gene, explaining why most of the *GhMDH* genes exhibited the relatively low expression levels under normal growth conditions (Figs 4 and 6). Likewise, the transcription level of *PgMDH* under chilling stress was maximum at 1 hour post-treatment and then decreased very little, while, during salinity stress (100 mM NaCl) the *PgMDH* was highly expressed and continues to increase from 1 day to 3 day treatment, indicated that this gene was related with response to stress and expression was increased after treatments of chilling and salt [78]. On the other hand, iTRAQ based proteomic analysis of cotton roots under salt stress concluded that there is a weak correlation between the transcript level of MDH gene and corresponding protein abundance, which highlights the effect of post-transcriptional modifications [79]. However, MDH genes, which are up-regulated during several abiotic stresses, are likely to be required to enhance resistance to stress, for example, the overexpression of *MdcyMDH* conferred high tolerance to cold and salt stresses in apple callus and transgenic tomatoes [8]. Therefore, we hypothesized that *GhMDHs* might also play essential roles in adaptation to a contrary environment. These data may indicate that the *cis*-elements in this study play important roles in regulating gene expression; further studies are required to confirm the relationship between the expression profiles of *GhMDHs* and the *cis*-regulatory elements in their promoter regions.

## Conclusions

In summary, our work led to the identification of 13 MDH genes *G*. *raimondii* and 25 *MDH* genes in *G*. *hirsutum*. The majority of the 51 cotton MDH members were dispersed as singletons. Our comparative analyses rendered worthy insight into the understanding of the phylogenetic relationships, sequence features, gene structure, motif composition and molecular evolution of MDH genes among the three cotton species. In addition, allotetraploid cotton *MDH* genes were differentially expressed in vegetative tissues and in fiber development stages from 0 DPA to 25 DPA, yielding a better understanding of possible functional divergence. Considered together, these results constitute a foundation for further studies, especially examining the functional characterization of the MDH gene family in cotton.

## Ethics Statement

No specific permits were required for the described field studies. The study involved the Asiatic diploid cultivated cotton plant (*G*. *hirsutum*), which is neither an endangered nor a protected species.

## Supporting Information

S1 Fig Sequence similarity percentage and domain structures of MDH genes from *G*. *arboretum*, *G*. *raimondii* and *G*. *hirsutum*. A. The sequence identities of cotton MDHs at both the nucleotide (below diagonal) and amino acid (above diagonal) levels from *G*. *arboretum*, *G*. *raimondii*, and *G*. *hirsutum*. The data on the diagonal lines are equal to 100%, and light blue and yellow color scale indicates the levels of identity at the top of the heat map. B. Domain architectures of cotton MDH proteins. All fifty-one cotton MDH proteins were analyzed for the presence of a functional domain (s) using SMART and Pfam (http://pfam.xfam.org/).
(TIF)

S2 Fig Motif logos of conserved motifs generated by MEME. (TIF)

S1 Table Nucleic acid, deduced amino acid, genomic and promoter sequence from sequences of the *GaMDH*, *GrMDH* and *GhMDH* genes. (XLSX)

S2 Table List of primers used for qRT-PCR and semi-quantitative PCR. (XLSX)

S3 Table Regular expression profile of the conserved motifs defined by MEME. (XLSX)

S4 Table Duplicated *GrMDH* and *GhMDH* genes and the number of conserved protein-coding genes flanking them. (XLSX)

S5 Table The *Ka/Ks* ratios for duplicated MDH genes in *G*. *arboretum*, *G*. *raimondii*, and *G*. *hirsutum*. (XLSX)

S6 Table Identification of putative *cis*-regulatory elements in the *G*. *arboretum*, *G*. *raimondii* and *G*. *hirsutum* malate dehydrogenase gene promoters. (XLSX)

S7 Table Sequence analyses of all putative GaMDH, GrMDH and GhMDH proteins for the presence of conserved motifs and enzyme activity. (XLSX)
