# Supplementary figures and images for: Comparative Genome-Wide Analysis of the Malate Dehydrogenase Gene Families in Cotton
Source: PLoS One. 2016 Nov 9;11(11):e0166341. doi: 10.1371/journal.pone.0166341 (PMC5102359; doi:10.1371/journal.pone.0166341)

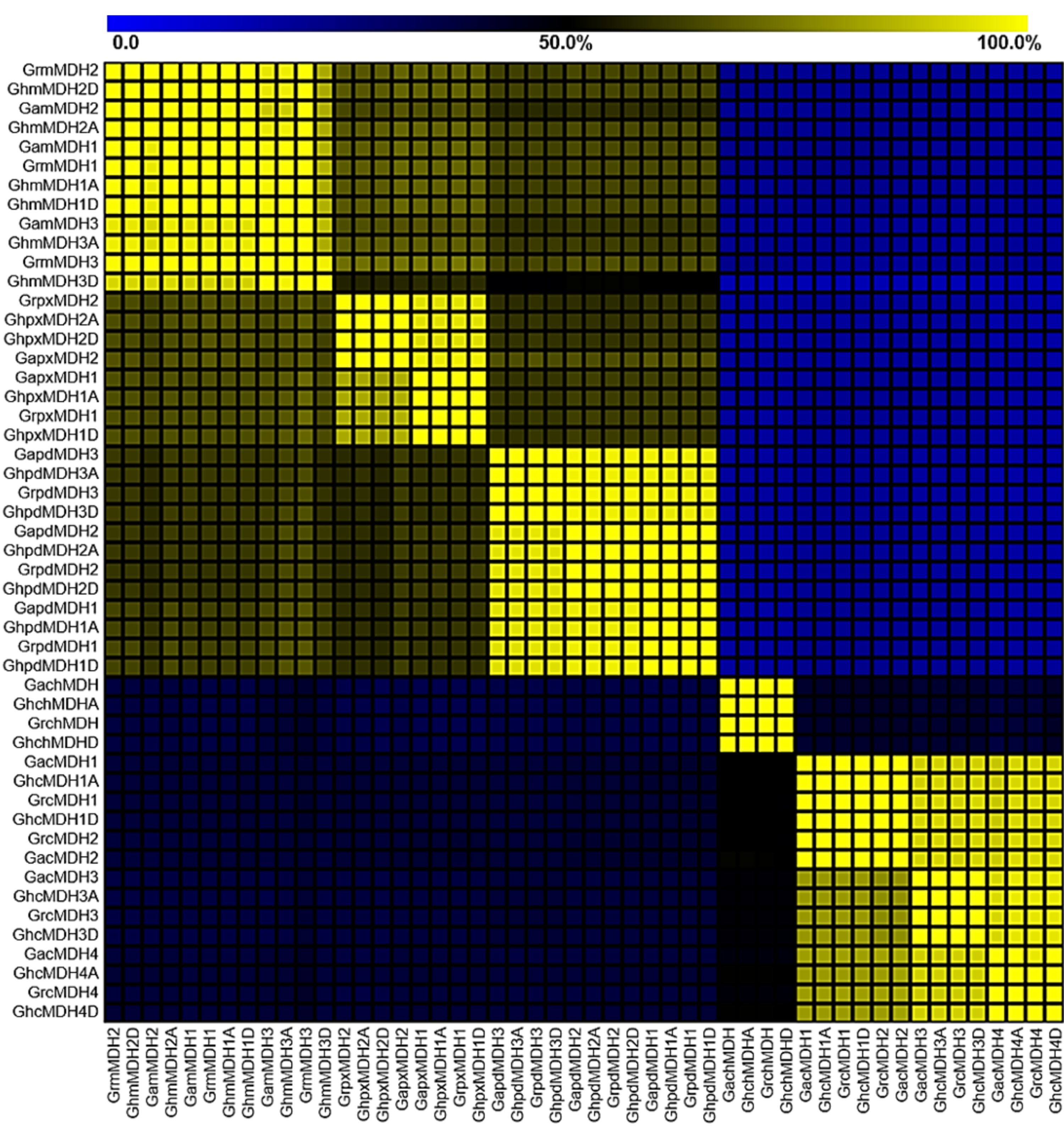

Supplement: S1 Fig — A. The sequence identities of cotton MDHs at both the nucleotide (below diagonal) and amino acid (above diagonal) levels from G. arboretum, G. raimondii, and G. hirsutum. The data on the diagonal lines are equal to 100%, and light blue and yellow color scale indicates the levels of identity at the top of the heat map. B. Domain architectures of cotton MDH proteins. All fifty-one cotton MDH proteins were analyzed for the presence of a functional domain (s) using SMART and Pfam (http://pfam.xfam.org/). (TIF) [file pone.0166341.s001.tif]

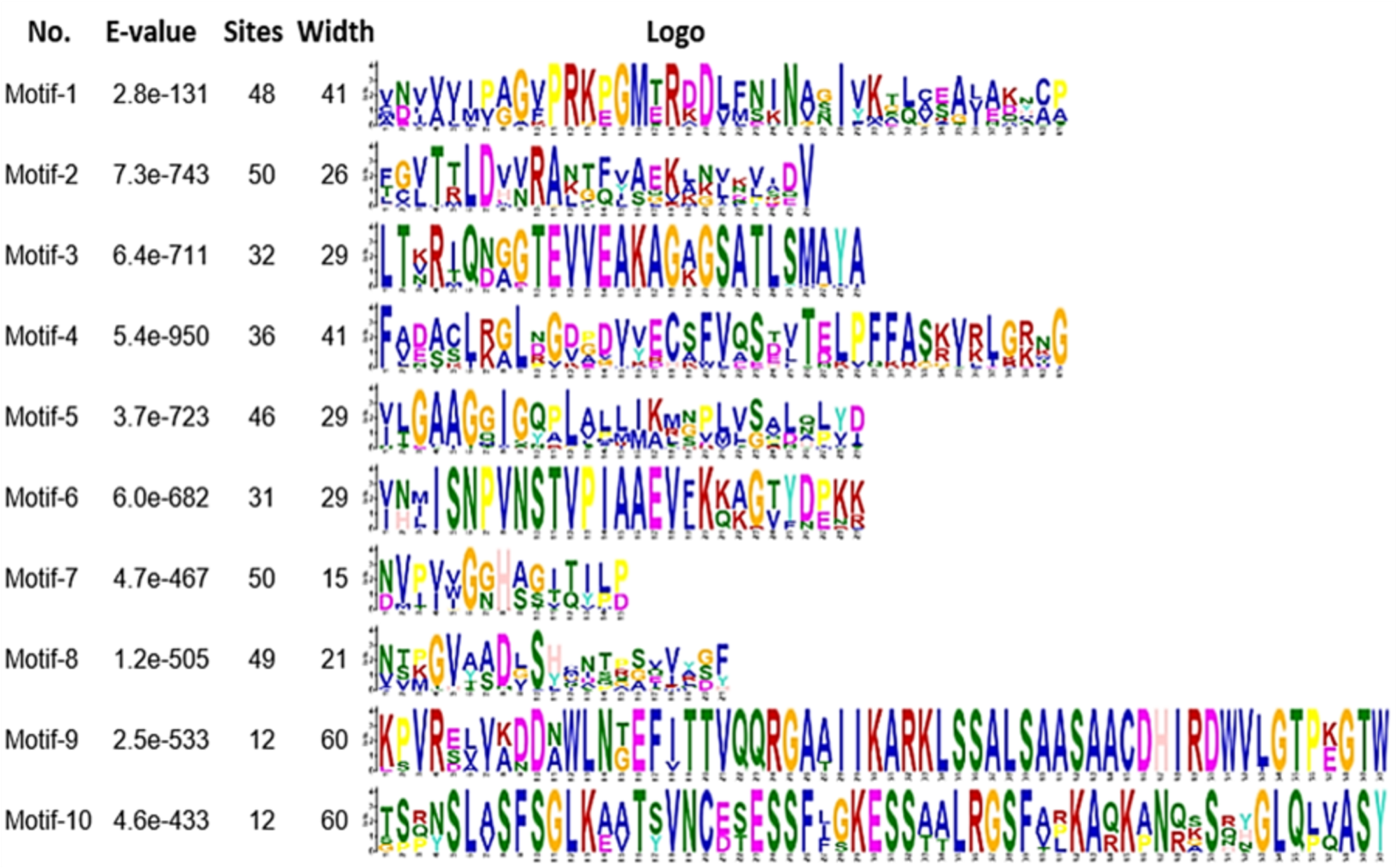

Supplement: S2 Fig — (TIF) [file pone.0166341.s002.tif]
